# Integrating child eye health within primary health care: a case study

**Published:** 2018-02-08

**Authors:** Milka Mafwiri, Aeesha NJ Malik, Clare Gilbert

**Affiliations:** 1 Senior Lecturer: Department of Ophthalmology, Muhimbili University of Health and Allied Sciences, Dar es Salaam, Tanzania.; 2 Clinical Research Fellow: International Centre for Eye Health, London School of Hygiene & Tropical Medicine, London, UK.; 3 Co-director: International Centre for Eye Health, London School of Hygiene & Tropical Medicine, London, UK.

**Few children have access to specialised eye care in Tanzania. However, ten simple activities have been shown to reduce the risk of childhood blindness and eye disease dramatically. By teaching these to primary health workers in Tanzania, thousands of babies now have a better chance of having healthy eyes.**

**Figure:**
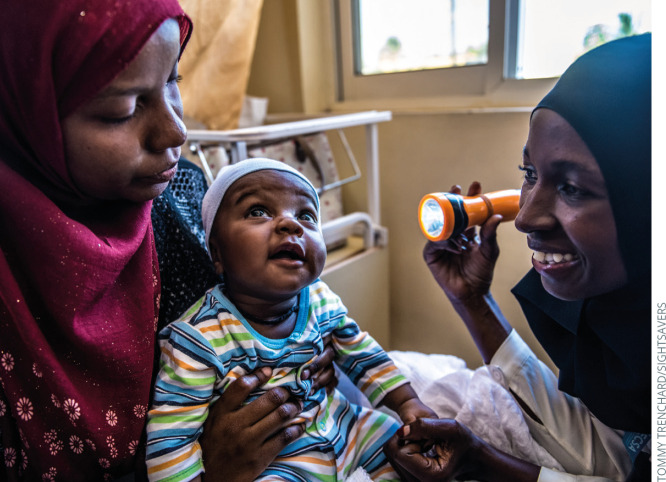
A baby with healthy eyes. ZANZIBAR

The control of blindness in children is a priority of VISION 2020, a global initiative with the goal of eliminating avoidable blindness by the year 2020. Many of the causes of visual loss in children in low-and middle-income countries are preventable; the most common of which are vitamin A deficiency, measles, conjunctivitis of the newborn and the use of harmful traditional remedies. Other causes of blindness, such as cataract, are treatable.^1^

Primary eye care has the potential to play a major role in reducing blindness in children by carrying out specific preventive measures and identifying and referring children with treatable conditions.^2^

The preventive measures include:

- Maintaining high coverage with measles immunisation and vitamin A supplementation
- Credé's prophylaxis to prevent ophthalmia neonatorum
- Health education regarding a good diet and breastfeeding
- Avoidance of traditional eye remedies

Early identification and referral of children is needed because, in many low- and middle-income countries (including Tanzania), children with cataract frequently present for surgery very late – often years after the parents first noticed the problem.^3–5^ Late presentation limits children's lifelong visual potential because seeing images clearly is important for their visual and general development when they are young.^6^ Some of the barriers to early presentation are beliefs that congenital blindness cannot be treated; being given the wrong advice by health workers; not knowing where to go; believing that the condition would resolve on its own and a preference for local remedies. Lack of education among mothers is a risk factor for late presentation.^4^

## Ten key activities to promote healthy eyes in children

In 2002, The World Health Organization (WHO) identified Ten key activities for healthy eyes' that would improve the eye health of children in Sub-Saharan Africa and South East Asia (Table 1).^7^ WHO recommended that these activities be implemented by primary health workers in reproductive and child health (RCH) clinics, the WHO'S Expanded Programme of Immunization, and the WHO'S Integrated Management of Childhood Illnesses programme.^8^

**Table 1:** Ten key activities to promote healthy eyes in children

**Control of general health conditions which can also lead to visual loss**	
Vitamin A deficiency	1. Give vitamin A supplements to children routinely
	2. Give vitamin A supplements to mothers after delivery
	3. Promote breastfeeding and good nutrition
Measles	4. Give vitamin A supplements to children with measles or malnutrition
	5. Immunise children against measles
**Control of eye conditions**	
Conjunctivitis of the newborn (ophthalmia neonatorum)	6. Clean the eyes of all babies at delivery and apply antibiotic eye drops or tetracycline eye ointment
Trachoma	7. Keep children's faces clean
Cataract	8. Refer children with poor vision or white pupils to an eye worker
Traditional eye remedies	9. Avoid the use of traditional eye medicines
Trauma	10. Refer children with history of injury to an eye worker

## Pilot study

In Tanzania, late presentation occurs despite the fact that most young children (up to 5 years of age) have frequent contact with trained health personnel in RCH clinics for growth monitoring, immunisation, treatment of different diseases and health education. These appointments were a missed opportunity to identify children with eye problems.

This prompted us to conduct a pilot study in 2010 in RCH clinics in urban Dar es Salaam. We were interested to find out if there were any changes in workers' knowledge, attitudes and practices before, and 12 months after, a one-day training session on the ten key activities for healthy eyes.

Before the training session, the key activities for healthy eyes which relate to general care (i.e. vitamin A supplementation and measles immunisation) were being routinely implemented in RCH clinics included in the study. However, the key activities which specifically relate to eye health (e.g. application of tetracycline after birth to prevent ophthalmia neonatorum) were not being implemented. After the training session, the RCH workers had better knowledge of eye conditions and changed some practices, such as cleaning the eyes of newborn babies at delivery and instilling an antibiotic or antiseptic, and referring children with trauma, a white pupil, or red eyes. Lack of knowledge, skills and supervision were the reasons given that staff members had failed to implement all of the eye-specific activities.^2^ It became clear that full and effective implementation of the ten key activities would not only control blindness, but also contribute towards reduction of under-five mortality rates.^9^

Following the study in Dar es Salaam, we worked with a steering committee to conduct further mixed methods research in RCH clinics and communities in the Singida region. Singida region is mainly rural and so more typical of Tanzania, compared to Dar es Salaam. The study, conducted in 2014, included facility surveys, observational checklists, interviews with staff, the assessment of case management of eye conditions using images, and interviews with key informants. A sample of mothers of children aged up to 24 months were also interviewed to assess their knowledge, awareness and health-seeking behaviour and to assess coverage of measles immunisation and vitamin A supplementation. The results were similar to those of the pilot study: most RCH workers had little knowledge of eye conditions, management of eye conditions was poor and ocular prophylaxis had been stopped or was inadequately implemented. Measles vaccines and vitamin A supplements were sometimes out of stock and health education sessions very rarely included eye conditions. Mothers did not know that measles immunisation and vitamin A can prevent blindness, and only a quarter of children aged 9–24 months had documented evidence of having received a vitamin A supplement on their ‘Road to Health’ charts. Measles immunisation coverage was good (84.7%).

**Figure:**
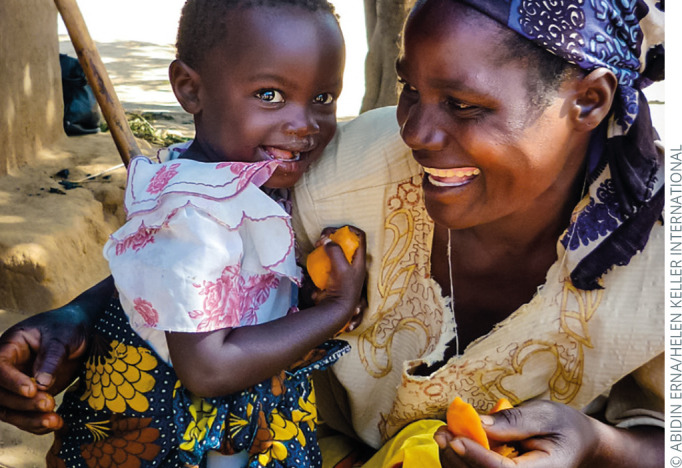
Having enough nutrient-rich food to eat prevents stunting, blindness, and death in young children.

## Integrated Management of Childhood Illness programme (IMCI)

When we explored a sustainable way to integrate primary eye care for children into primary health services, we considered the Integrated Management of Childhood Illness programme (IMCI). IMCI was developed by UNICEF and WHO to reduce morbidity and mortality in children aged 0–5 years. The programme has three elements:

1. Improving partnerships between health facilities or services and the communities they serve
2. Increasing appropriate and accessible care and information from community-based providers
3. Integrating promotion of key family practices critical for child health and nutrition.

IMCI has been adopted and implemented with varying degrees of success in low- and middle-income countries, including Tanzania.^10^ Although it was developed as a comprehensive approach, the focus has been on treatment within health facilities. Tanzania is among the countries that have embraced IMCI and the programme is being implemented through the RCH services. In Tanzania, IMCI training is delivered in two ways: pre-service training and in-service training involving ten modules covering different conditions. Notably, IMCI has a section on ear health but there is no section dedicated to eye health.

## Eye health module in IMCI

In April 2017, findings from both studies were presented to the steering committee in Tanzania, which included ministry of health officials responsible for IMCI. The committee agreed that an eye module should be added to the national IMCI in-service training package. The eye module has since been developed in collaboration with the ministry of health. A poster has been designed and DVD clips are being prepared on topics such as how to examine the eyes of a young child. All these materials will be tested and then they will be ready for use in the national IMCI programme. As training is being rolled out, we plan to undertake an evaluation to assess the effectiveness of this training on how RCH staff manage eye conditions in children and to assess whether it has improved other aspects of control, such as vitamin A supplementation coverage.

## Conclusion

Inclusion of the eye module in IMCI in Tanzania is a significant step forward and has the potential to substantially reduce blindness in children. Findings from the planned evaluation will be used in advocacy so that other countries in Africa may include eye conditions in their IMCI training package.

## Acknowledgements

The studies were supported by grants from Task Force Sight and Life, Sightsavers, the British Council for the Prevention of Blindness and CBM.

